# Supplementary material for: Adenine base editing efficiently restores the function of Fanconi anemia hematopoietic stem and progenitor cells
Source: Nat Commun. 2022 Nov 12;13:6900. doi: 10.1038/s41467-022-34479-z (PMC9653444; doi:10.1038/s41467-022-34479-z)
Supplement: Supplementary file 1 — supplementary information [file 41467_2022_34479_MOESM1_ESM.pdf]

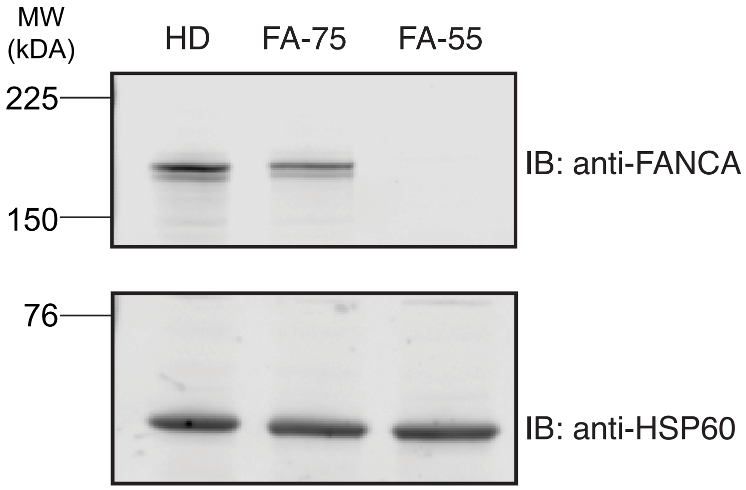

Supplementary Fig. 1: FANCA protein detection by Western blot.

Protein extracts were analyzed by Western blotting and probed with anti-FANCA and anti-HSP60. n=2, for the biological independent experiments.

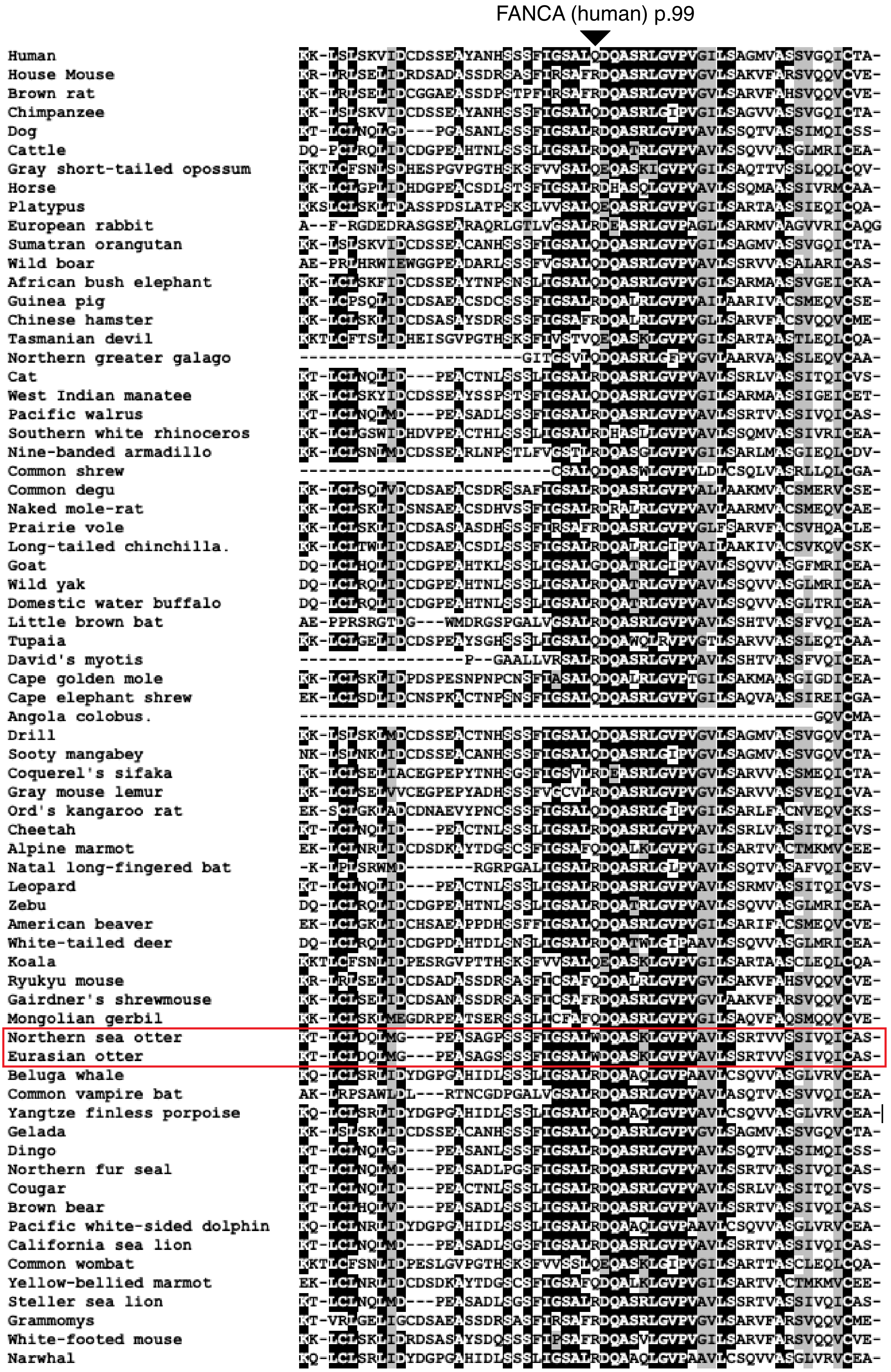

Supplementary Fig. 2: FANCA protein alignment in a great number of vertebrate species.

Northern sea otter and Eurasian otter alignment were highlighted with the red rectangle, indicating the presence of W (Tryptophan) corresponding at the aminoacid position 99 of human FANCA.

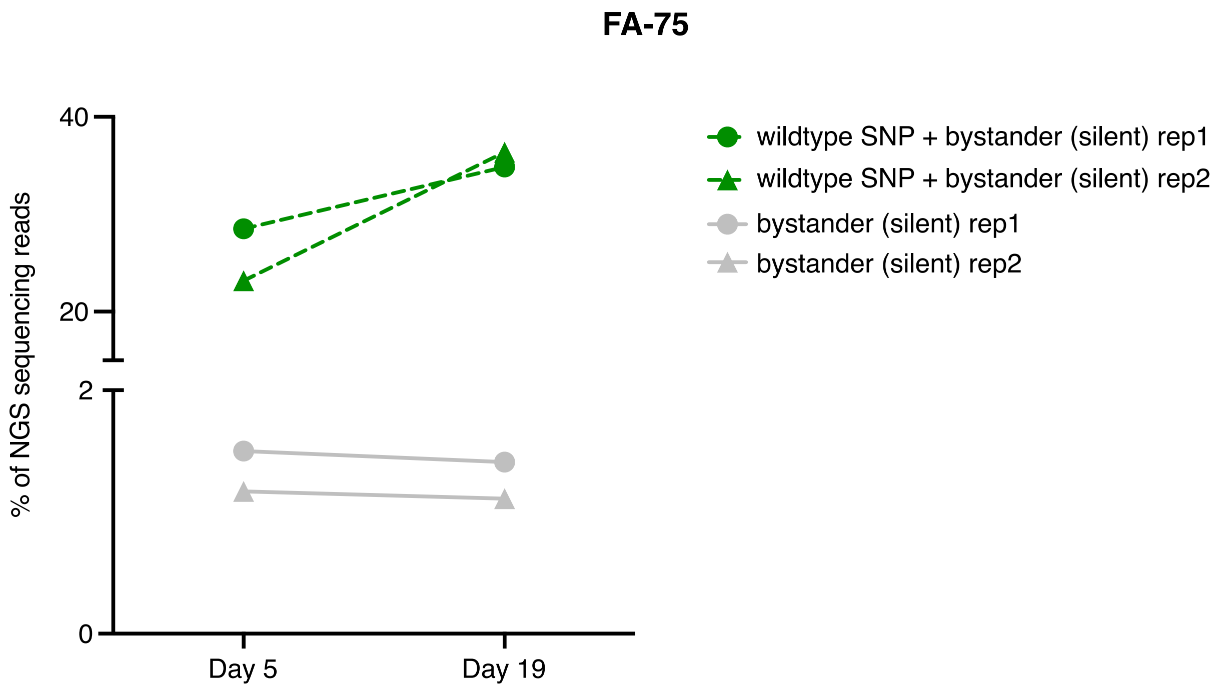

Supplementary Fig. 3: Expansion of sequencing reads with the wildtype SNP but not with the bystander alone.

Sequencing reads of FA-75 editing with mABE8e + sgRNA4s in Figure 1C containing the wildtype SNP and the silent bystander or the bystander alone were analyzed for their abundance in the sequencing pool. From Day 5 to Day 19 the sequencing reads containing the wildtype SNP increased, while the reads only containing the silent bystanderwere slightly decreasing in both replicates.

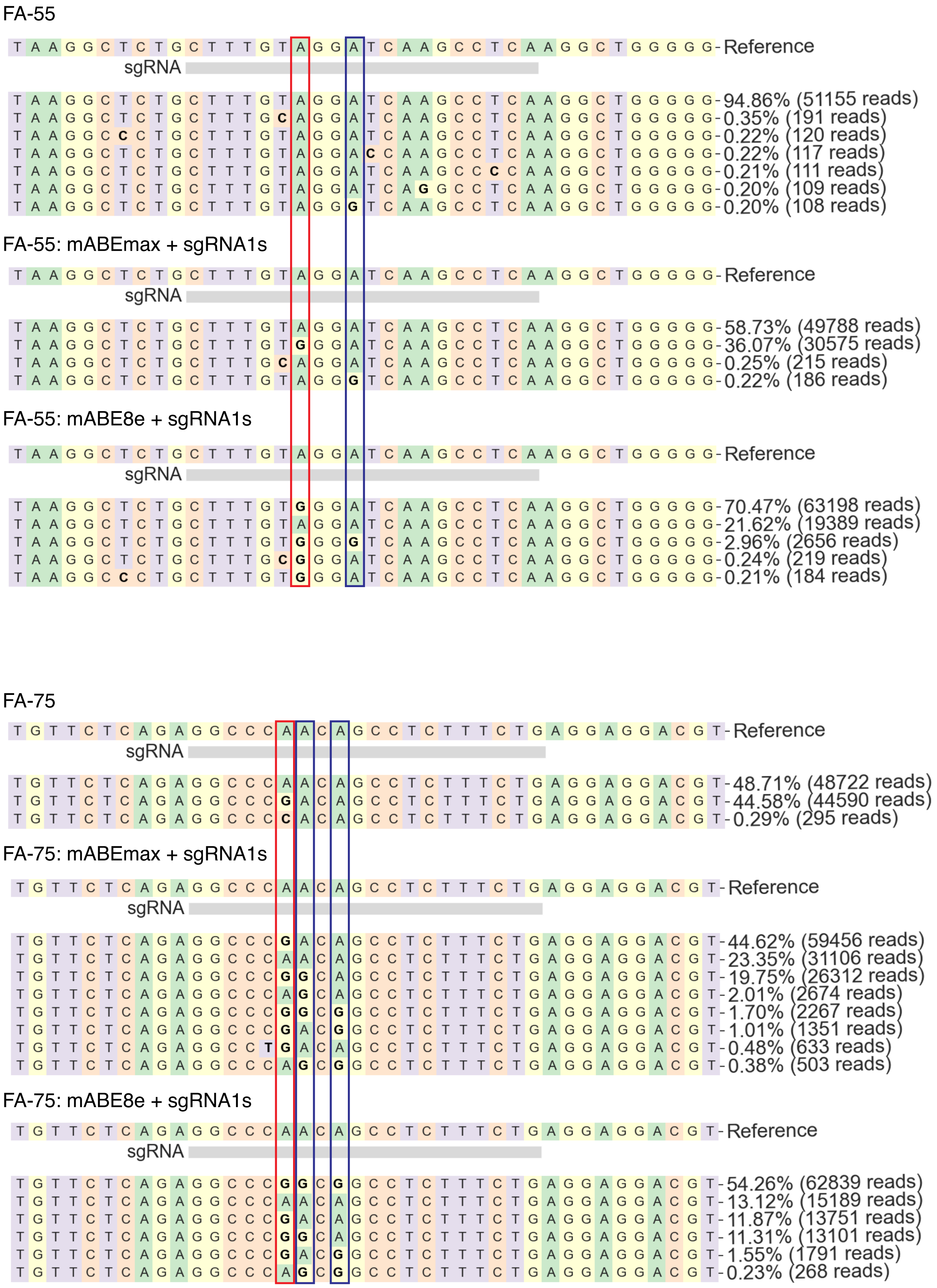

Supplementary Fig. 4: Allele frequency tables for FA-55 and FA-75 editing with mABEmax and ABE8e.

Allele frequency tables around the sgRNAs from CRISPResso2 are shown for all editing scenarios in Figure 3. We saw very high on-target editing in FA-55 and FA-75 (red rectangle). Bystander editing (blue rectangles) was high in sgRNA4 in FA-75 with either ABEmax or ABE8e, while in FA-55 we only detected

FA-55

```
CTTTGTAGGATCAAGCCTCAAGG ON
..........GT........G.. OT1
......GA.......T....G.. OT2
...........A.....AA.T.. OT3
.....G......C....C..... OT4
.....A......G.T.....T.. OT5
.......TC..A........T.. OT6
........TC...G......... OT7
G..........A..T........ OT8
```

FA-75

```
GGCCCAACAGCCTCTTTCTGAGG ON
T.....C...G............ OT1
T.....C...G............ OT2
....GC.T............... OT3
.T....G....A........... OT4
...T..T.....C.......... OT5
T......A.....G......... OT6
..T........T..C........ OT7
..........GT.A......... OT8
```

Supplementary Fig. 5: Top Cas-OFFinder off-target sites for FA-55 sgRNA1 and FA-75 sgRNA4

Computationally predicted off-target sites are shown for FA-55 sgRNA1 and FA-75 sgRNA4. Dots represent base identical to the on-target locus. PAM sites of FA-55 sgRNA1 and FA-75 sgRNA4 are underlined.

a
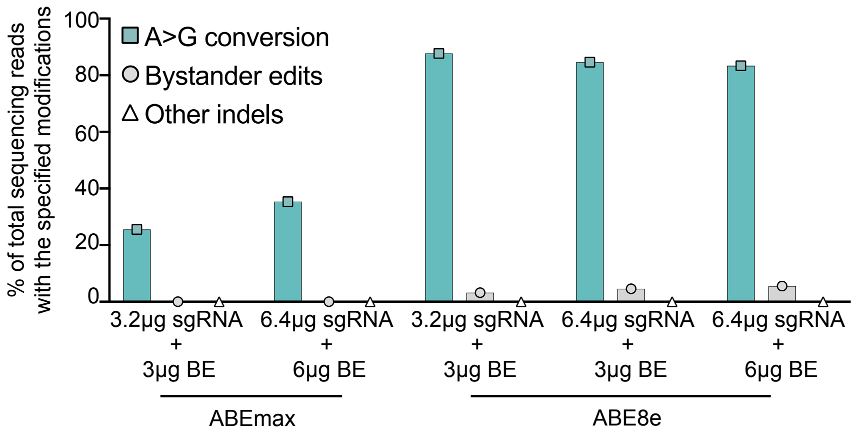

b
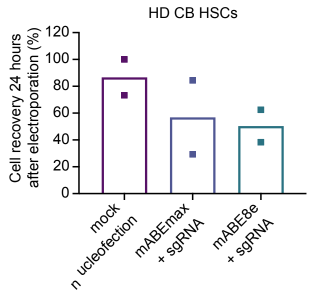

c
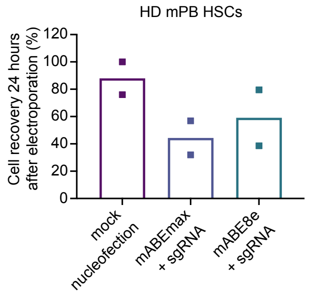

d
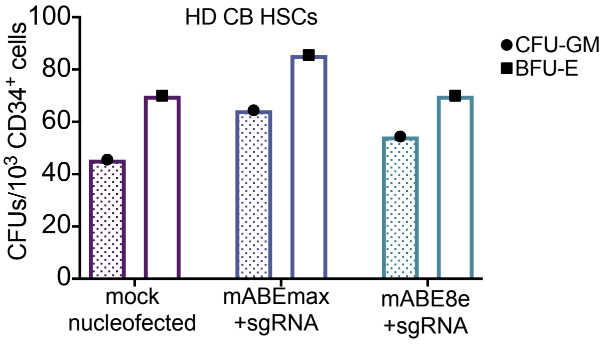

ABEmax BE HD CB CFUs

Allele	Editing	Sequence	Frequency
1	Biallelic	TGT**CCC**T**G**G**TGG**CCC**CAC**TGT	33.33% (1/3)
2		TGT**CCC**T**G**G**TGG**CCC**CAC**TGT	
1	Monoalelic	TGT**CCC**T**G**G**TGG**CCC**CAC**TGT	66.66% (2/3)
2		TGT**CCC**T**A**G**TGG**CCC**CAC**TGT	

ABE8e BE HD CB CFUs

Allele	Editing	Sequence	Frequency
1	Biallelic	TGT**CCC**T**G**G**TGG**CCC**CAC**TGT	100% (15/15)
2		TGT**CCC**T**G**G**TGG**CCC**CAC**TGT	
1	Monoalelic	TGT**CCC**T**G**G**TGG**CCC**CAC**TGT	0%
2		TGT**CCC**T**A**G**TGG**CCC**CAC**TGT	

e
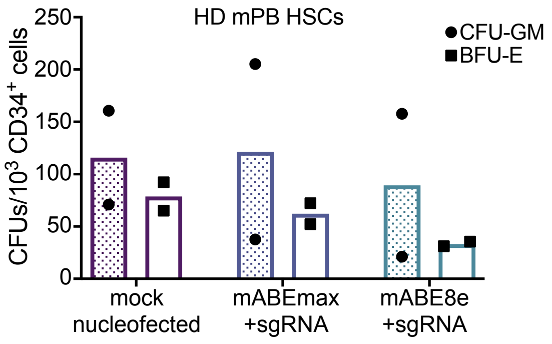

ABEmax BE HD mPB CFUs

Allele	Editing	Sequence	Frequency
1	Biallelic	TGT**CCC**T**G**G**TGG**CCC**CAC**TGT	0%
2		TGT**CCC**T**G**G**TGG**CCC**CAC**TGT	
1	Monoalelic	TGT**CCC**T**G**G**TGG**CCC**CAC**TGT	100% (3/3)
2		TGT**CCC**T**A**G**TGG**CCC**CAC**TGT	

ABE8e BE HD mPB CFUs

Allele	Editing	Sequence	Frequency
1	Biallelic	TGT**CCC**T**G**G**TGG**CCC**CAC**TGT	100% (13/13)
2		TGT**CCC**T**G**G**TGG**CCC**CAC**TGT	
1	Monoalelic	TGT**CCC**T**G**G**TGG**CCC**CAC**TGT	0%
2		TGT**CCC**T**A**G**TGG**CCC**CAC**TGT	

f
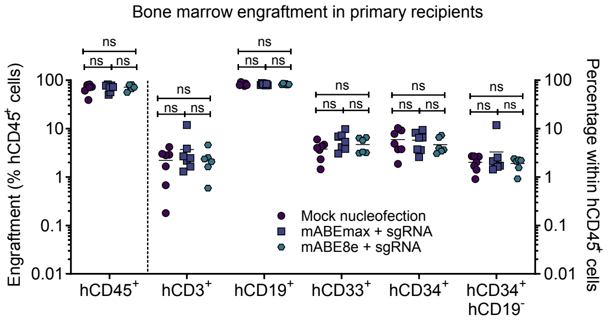

g
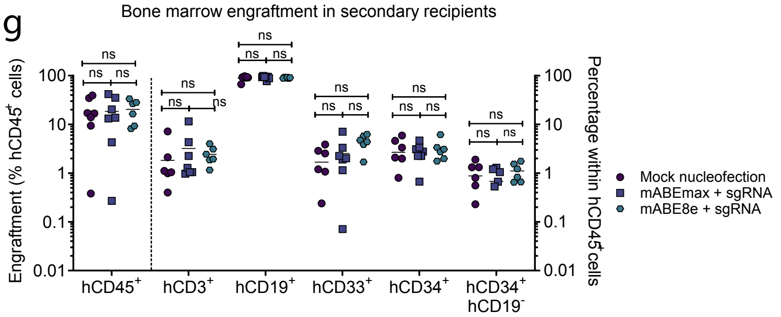

h
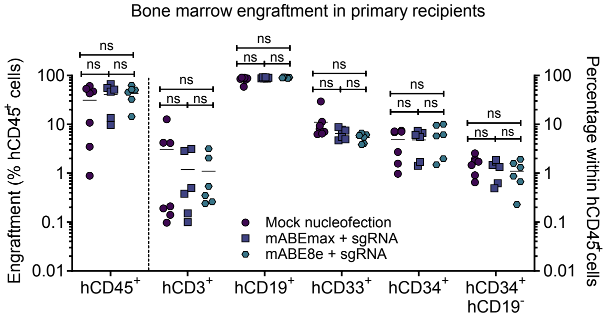

Supplemental Fig. 6: Adenine base editing efficiency in healthy donor (HD) $CD34^+$ cells.

a) Optimization of gene editing efficiencies using mABEmax and mABE8e base editors in HD CB $CD34^+$ cells. Data correspons to 1 experiment.
b and c) CB and mPB cell recovery analyzed 24 hours after mock, mABEmax and mABE8e mRNA and synthetic gRNA electroporation. Bars represent mean value from two independent experiments; error bars indicate the range. Data correspond to cells shown in Fig. 5b and c.
d and e) Upper: number of hematopoietic myeloid and erythroid colonies (CFU-GM and BFU-E, respectively) per $1x10^3$ HD $CD34^+$ cells after mock, mABEmax and mABE8e mRNA and synthetic gRNA electroporation in CB and mPB $CD34^+$ cells. Lower: Analysis of the frequency of specific editing events in edited human hematopoietic colonies (Total CFUs: CFUGM + BFU-E). Bars represent mean value of three experimental replicates from one independent experiment (d) and two independent experiments (e).Data correspond to CFUs obtained from cells shown in Fig. 5b and c.
f and g) Multilineage repopulation of human HD CB $CD34^+$ cells in bone marrow from primary and secondary recipient mice 90 days post-transplant. Mean values are represented with a horizontal bar (number of mice analyzed: n=7, n=7 and n=6 in primary and secondary recipients at 90 days for mock, mABEmax and mABE8e, respectively). In all cases, a two-way ANOVA was performed followed by a Tukey's multiple comparison test: ns = not significant.
h) Multilineage repopulation of human HD mPB $CD34^+$ cells in bone marrow from primary recipient mice 90 or 120 days post-transplant (number of mice analyzed: n=7, n=7 and n=6 for mock, mABEmax and mABE8e. Mean values are represented with a horizontal bar. In all cases, a two-way ANOVA was performed followed by a Tukey's multiple comparison test: ns = not significant.

a

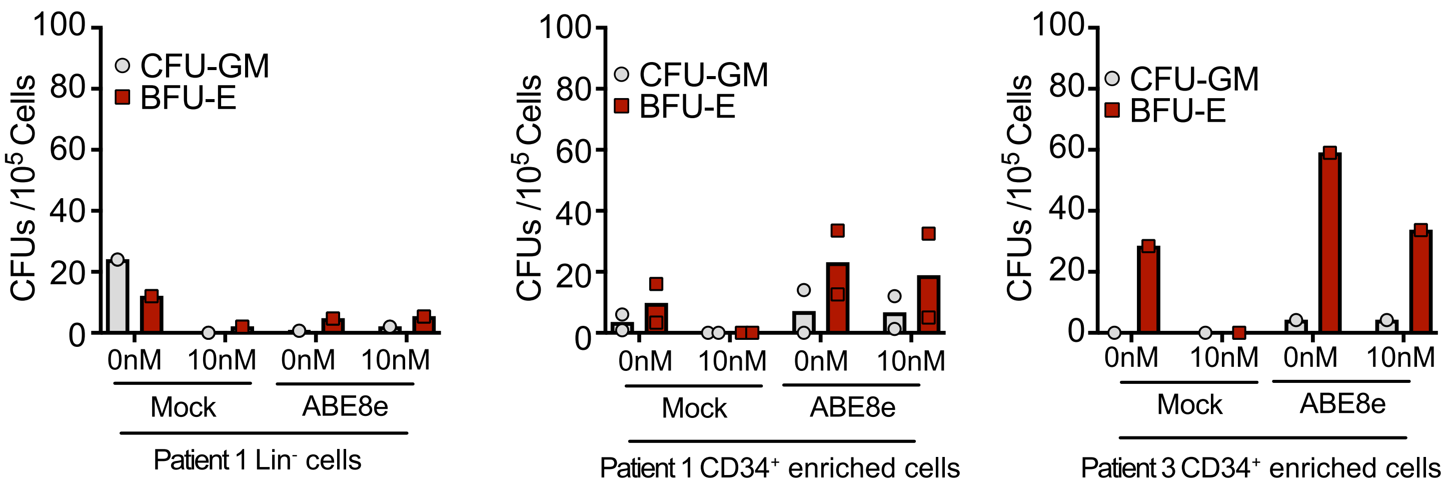

b

ABE8e BE Patient 1 Lin⁻ CFUs 0nM MMC

Allele	Editing	Sequence	Frequency
1	Biallelic	TCTGCTTTGTGGGATCAAGCC	100% (9/9)
2		TCTGCTTTGTGGGATCAAGCC	
1	Monoallelic	TCTGCTTTGTGGGATCAAGCC	0% (0/9)
2		TCTGCTTTGTAGGATCAAGCC	

ABE8e BE Patient 1 CD34⁺ enriched CFUs 0nM MMC

Allele	Editing	Sequence	Frequency
1	Biallelic	TCTGCTTTGTGGGATCAAGCC	84.62% (22/26)
2		TCTGCTTTGTGGGATCAAGCC	
1	Monoallelic	TCTGCTTTGTGGGATCAAGCC	15.38% (4/26)
2		TCTGCTTTGTAGGATCAAGCC	

ABE8e BE Patient 1 Lin⁻ CFUs 10nM MMC

Allele	Editing	Sequence	Frequency
1	Biallelic	TCTGCTTTGTGGGATCAAGCC	100% (11/11)
2		TCTGCTTTGTGGGATCAAGCC	
1	Monoallelic	TCTGCTTTGTGGGATCAAGCC	0% (0/11)
2		TCTGCTTTGTAGGATCAAGCC	

ABE8e BE Patient 1 CD34⁺ enriched CFUs 10nM MMC

Allele	Editing	Sequence	Frequency
1	Biallelic	TCTGCTTTGTGGGATCAAGCC	94.12% (32/34)
2		TCTGCTTTGTGGGATCAAGCC	
1	Monoallelic	TCTGCTTTGTGGGATCAAGCC	5.88% (2/34)
2		TCTGCTTTGTAGGATCAAGCC	

ABE8e BE Patient 2 CD34⁺ enriched CFUs 0nM MMC

Allele	Editing	Sequence	Frequency
1	Biallelic	TCTGCTTTGTGGGATCAAGCC	89.19% (33/37)
2		TCTGCTTTGTGGGATCAAGCC	
1	Monoallelic	TCTGCTTTGTGGGATCAAGCC	10.81% (4/37)
2		TCTGCTTTGTAGGATCAAGCC	

ABE8e BE Patient 3 CD34⁺ enriched CFUs 0nM MMC

Allele	Editing	Sequence	Frequency
1	Biallelic	TCTGCTTTGTGGGATCAAGCC	100% (6/6)
2		TCTGCTTTGTGGGATCAAGCC	
1	Monoallelic	TCTGCTTTGTGGGATCAAGCC	0%
2		TCTGCTTTGTAGGATCAAGCC	

ABE8e BE Patient 3 CD34⁺ enriched CFUs 10nM MMC

Allele	Editing	Sequence	Frequency
1	Biallelic	TCTGCTTTGTGGGATCAAGCC	100% (7/7)
2		TCTGCTTTGTGGGATCAAGCC	
1	Monoallelic	TCTGCTTTGTGGGATCAAGCC	0%
2		TCTGCTTTGTAGGATCAAGCC	

Supplementary Fig. 7: Clonogenic capacity of mABE8e edited cells and analysis of the editing outcome in individual colonies.

a) Number of hematopoietic myeloid and erythroid colonies (CFU-GM and BFU-E, respectively) per 10^5 CD34⁺ enriched cells from three FA-A patients after mock and mABE8e electroporation in the absence (0nM) and presence (10nM) of MMC selection. Bars indicate the value from one experiment patient 1 Lin⁻ cells and mean values of two independent experiments in patient 1 CD34⁺ enriched cells. Bars indicate the value from one experiment in patient 3 CD34⁺ enriched cells. Indiviual dots represent the measurements in each experiment. Error bars indicate SD.
b) Frequency of monoallelic and biallelic A>G therapeutic events in edited FA-A individual CFUs. Frequency shows the percentage of edited CFUs analyzed with the specified genotype and the number of CFUs analyzed (Total CFUs: CFU-GM + BFU-E).

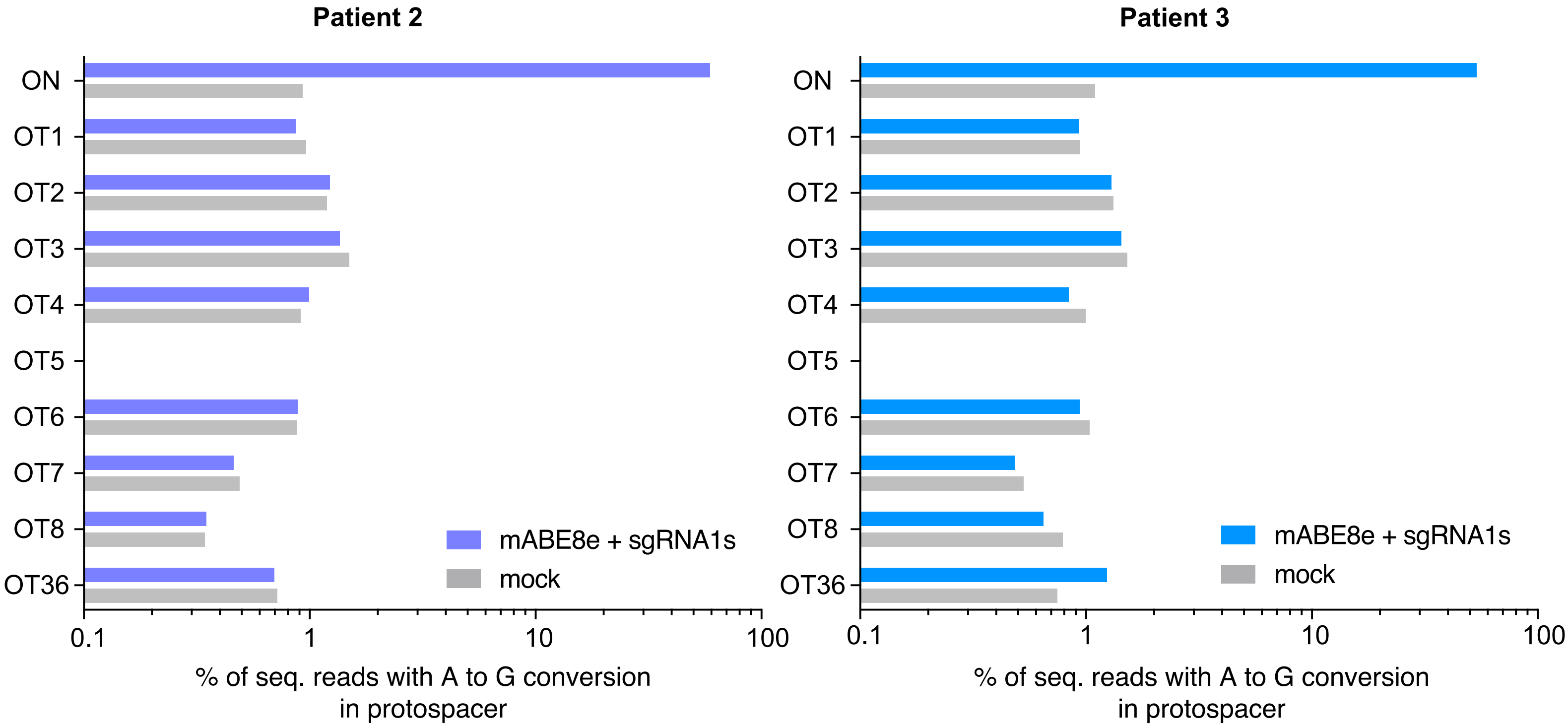

Supplementary Fig. 8: Off-target analysis in FA patient HSPCs (patient 2 and 3) edited with mABE8e and sgRNA1s.

Off target sites were selected corresponding to FA-55 sgRNA1 sites (Figure 4 & Supplementary Figure 5). Genomic loci were amplified and subjected to next generation sequencing. Sequencing reads with one or more A to G conversions in the protospacer were plotted for the each gRNA. At OT5, a homozygous A to G SNP was found in both patients. None of the the investigated sites showed significant higher A to G conversion than background levels.

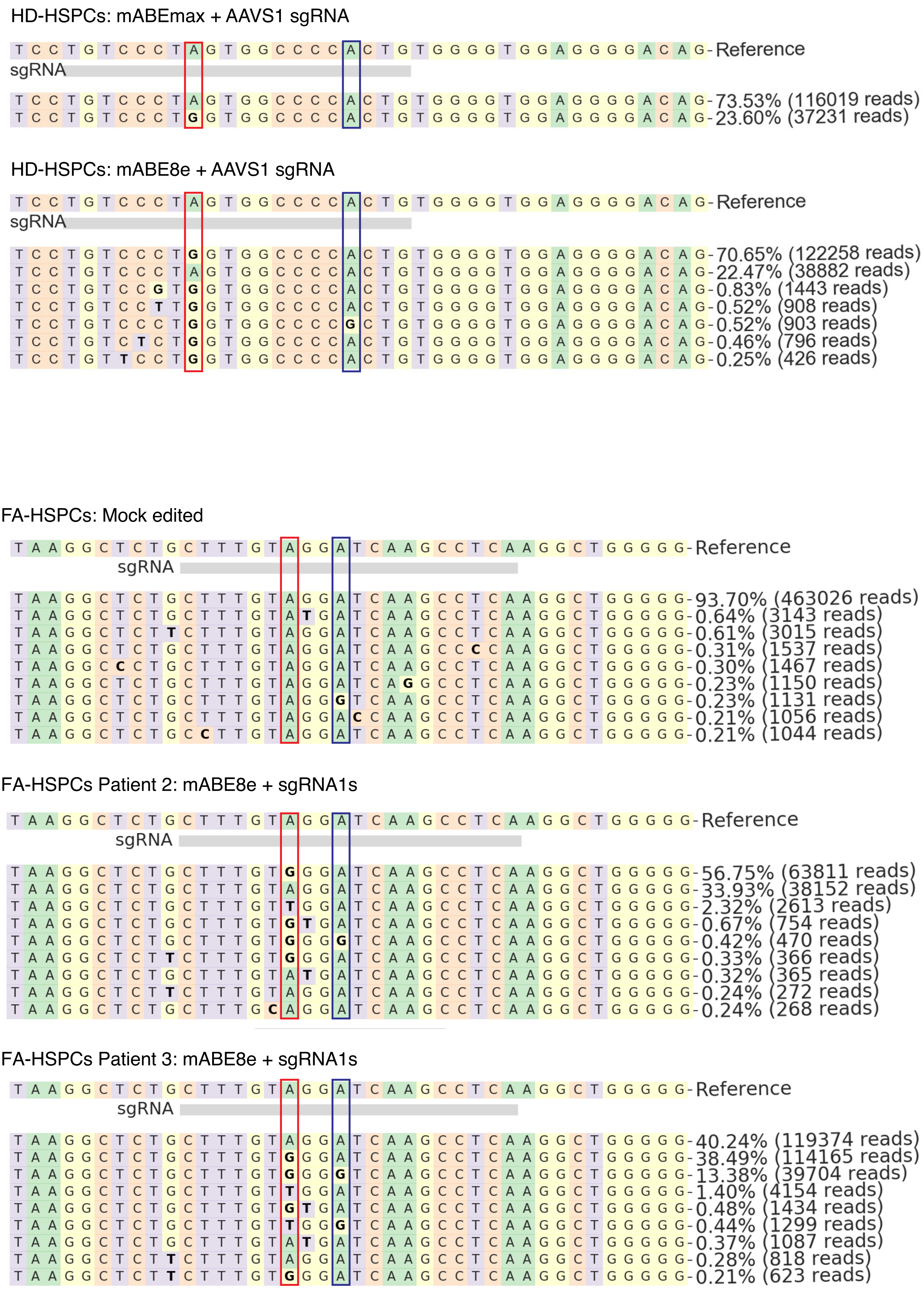

Supplementary Fig. 9: Allele frequency tables for editing AAVS1 and FA patient HSPCs (FANCA c.295C>T).

Allele frequency tables around the sgRNAs from CRISPResso2 output are shown for editing scenarios in Figure 5 and 6. We observed moderate on-target editing in AAVS1 with mABEmax and high editing efficiency with mABE8e (red rectangle) with few bystander events (blue rectangle). Editing patient 2 and 3 HSPCs with mABE8e and sgRNA1 resulted in high level on-targeting editing (red rectangle). Bystander editing was higher in patient 3 than 2 (blue rectangle).

Supplementary Figure 10

OT analysis

2022-09-09

Analysis script for off-targets in FA LCLs and HSPCs for Figure 4, Supplementary Figure 5 and 8:

Allele frequency tables are derived from the CRISPResso2 output.

```
## 9th of September, 2022

library(stringr)

## FA HSPCs (sgRNA1 (FA55))

setwd("/Users/sebastiansiegner/Documents/Arbeit/Corn Lab data/Corn Lab/PhD-Co
rn/FA Base editing Paper/NatureComm Submission/Off Target analysis/CRISPResso
/FA55/FA HSPCs/Allele frequency tables/")

nuc_change_tab <- read.csv("Nucleotide_change_table.csv")

OT_quantification <- c("ABE8e_FA5","ABE8e_FA6","mock_FA5","mock_FA6")

for (i in 1:9)
{
  temp_change <- nuc_change_tab[i,2]
  temp_unaffected <- nuc_change_tab[i,3]
  temp_OT_name <- paste("OT",i,sep = "")
  OT_quantification_samples <- data.frame(c("ABE8e_FA5","ABE8e_FA6","mock_FA5
","mock_FA6"))

  for (k in 1:4)
  {
    temp_sample_name <-  paste("OT",i,"_",k,".txt",sep = "")
    temp_OT <- read.table(temp_sample_name, skip = 1)
    temp_OT <- temp_OT[,c(1,2,8)]
    temp_OT[,1] <- substr(temp_OT[,1],11,30)
    temp_OT[,2] <- substr(temp_OT[,2],11,30)
    temp_OT_variants <- temp_OT[2,2]
    temp_OT <- temp_OT[which((str_count(temp_OT[[1]],substr(temp_change,1,1))
< str_count(temp_OT_variants,substr(temp_change,1,1))) &
                               (str_count(temp_OT[[1]],substr(temp_change,3,3
)) > str_count(temp_OT_variants,substr(temp_change,3,3))) &
                               (str_count(temp_OT[[1]],substr(temp_unaffected
```

```
,1,1)) == str_count(temp_OT_variants,substr(temp_unaffected,1,1))) &
                               (str_count(temp_OT[[1]],substr(temp_unaffected
,3,3)) == str_count(temp_OT_variants,substr(temp_unaffected,3,3)))),]
    temp_off_target_editing <- colSums(temp_OT[3])
    OT_quantification_samples[k,2] <- temp_off_target_editing
  }
colnames(OT_quantification_samples) <- c("sample",temp_OT_name)
OT_quantification <- cbind(OT_quantification, OT_quantification_samples[2])
}

# On-Target

for (k in 1:4)
{
  temp_sample_name <-  paste("ON","_",k,".txt",sep = "")
  temp_OT <- read.table(temp_sample_name, skip = 1)
  temp_OT <- temp_OT[,c(1,2,8)]
  temp_OT[,1] <- substr(temp_OT[,1],11,30)
  temp_OT[,2] <- substr(temp_OT[,2],11,30)
  temp_OT_variants <- temp_OT[2,2]
  temp_OT <- temp_OT[which((str_count(temp_OT[[1]],"A") < str_count(temp_OT_v
ariants,"A")) &
                             (str_count(temp_OT[[1]],"G") > str_count(temp_OT
_variants,"G")) &
                             (str_count(temp_OT[[1]],"C") == str_count(temp_O
T_variants,"C")) &
                             (str_count(temp_OT[[1]],"T") == str_count(temp_O
T_variants,"T"))),]
  temp_off_target_editing <- colSums(temp_OT[3])
  OT_quantification_samples[k,2] <- temp_off_target_editing
  colnames(OT_quantification_samples) <- c("sample","ON")
}

OT_quantification_per <- cbind(OT_quantification, OT_quantification_samples[2
])
write.csv(OT_quantification_per, file = "OT_quantification_FA55_HSPCs.csv")

## FA75

setwd("/Users/sebastiansiegner/Documents/Arbeit/Corn Lab data/Corn Lab/PhD-Co
rn/FA Base editing Paper/NatureComm Submission/Off Target analysis/CRISPResso
/FA75/CRISPResso output/Allele frequency tables/")

nuc_change_tab <- read.csv("Nucleotide_change_table.csv")
order <- nuc_change_tab[,1]

j <- 0
OT_quantification <- c("ABE8e_n1","ABE8e_n2","ABEmax_n1","ABEmax_n2","BE_mRNA
_only_n1","BE_mRNA_only_n2")
```

```
for (i in order)
{
  j <- j +1
  temp_change <- nuc_change_tab[j,2]
  temp_unaffected <- nuc_change_tab[j,3]
  temp_OT_name <- paste("OT",i,sep = "")
  OT_quantification_samples <- data.frame(c("ABE8e_n1","ABE8e_n2","ABEmax_n1"
,"ABEmax_n2","BE_mRNA_only_n1","BE_mRNA_only_n2"))

  for (k in 1:6)
  {
    temp_sample_name <-  paste("OT",i,"_",k,".txt",sep = "")
    temp_OT <- read.table(temp_sample_name, skip = 1)
    temp_OT <- temp_OT[,c(1,2,8)]
    temp_OT[,1] <- substr(temp_OT[,1],11,30)
    temp_OT[,2] <- substr(temp_OT[,2],11,30)
    temp_OT_variants <- temp_OT[2,2]
    temp_OT <- temp_OT[which((str_count(temp_OT[[1]],substr(temp_change,1,1))
< str_count(temp_OT_variants,substr(temp_change,1,1))) &
                               (str_count(temp_OT[[1]],substr(temp_change,3,3
)) > str_count(temp_OT_variants,substr(temp_change,3,3))) &
                               (str_count(temp_OT[[1]],substr(temp_unaffected
,1,1)) == str_count(temp_OT_variants,substr(temp_unaffected,1,1))) &
                               (str_count(temp_OT[[1]],substr(temp_unaffected
,3,3)) == str_count(temp_OT_variants,substr(temp_unaffected,3,3)))),]
    temp_off_target_editing <- colSums(temp_OT[3])
    OT_quantification_samples[k,2] <- temp_off_target_editing
  }
  colnames(OT_quantification_samples) <- c("sample",temp_OT_name)
  OT_quantification <- cbind(OT_quantification, OT_quantification_samples[2])
}

# On-Target

for (k in 1:6)
{
  temp_sample_name <-  paste("ON","_",k,".txt",sep = "")
  temp_OT <- read.table(temp_sample_name, skip = 1)
  temp_OT <- temp_OT[,c(1,2,8)]
  temp_OT[,1] <- substr(temp_OT[,1],11,30)
  temp_OT[,2] <- substr(temp_OT[,2],11,30)
  temp_OT_variants <- temp_OT[2,2]
  temp_OT <- temp_OT[which((str_count(temp_OT[[1]],"A") < str_count(temp_OT_v
ariants,"A")) &
                             (str_count(temp_OT[[1]],"G") > str_count(temp_OT
_variants,"G")) &
                             (str_count(temp_OT[[1]],"C") == str_count(temp_O
T_variants,"C")) &
                             (str_count(temp_OT[[1]],"T") == str_count(temp_O
T_variants,"T"))),]
```

```
  temp_off_target_editing <- colSums(temp_OT[3])
  OT_quantification_samples[k,2] <- temp_off_target_editing
  colnames(OT_quantification_samples) <- c("sample","ON")
}

OT_quantification_per <- cbind(OT_quantification, OT_quantification_samples[2
])
write.csv(OT_quantification_per, file = "OT_quantification_75.csv")

## FA55

setwd("/Users/sebastiansiegner/Documents/Arbeit/Corn Lab data/Corn Lab/PhD-Co
rn/FA Base editing Paper/NatureComm Submission/Off Target analysis/CRISPResso
/FA55/CRISPResso2 output/Allele frequency table/")

nuc_change_tab <- read.csv("Nucleotide_change_table.csv")

OT_quantification <- c("ABE8e_n1","ABE8e_n2","ABEmax_n1","ABEmax_n2","BE_mRNA
_only_n1","BE_mRNA_only_n2")
for (i in 1:40)
{
  temp_change <- nuc_change_tab[i,2]
  temp_unaffected <- nuc_change_tab[i,3]
  temp_OT_name <- paste("OT",i,sep = "")
  OT_quantification_samples <- data.frame(c("ABE8e_n1","ABE8e_n2","ABEmax_n1"
,"ABEmax_n2","BE_mRNA_only_n1","BE_mRNA_only_n2"))

  for (k in 1:6)
  {
    temp_sample_name <-  paste("OT",i,"_",k,".txt",sep = "")
    temp_OT <- read.table(temp_sample_name, skip = 1)
    temp_OT <- temp_OT[,c(1,2,8)]
    temp_OT[,1] <- substr(temp_OT[,1],11,30)
    temp_OT[,2] <- substr(temp_OT[,2],11,30)
    temp_OT_variants <- temp_OT[2,2]
    temp_OT <- temp_OT[which((str_count(temp_OT[[1]],substr(temp_change,1,1))
< str_count(temp_OT_variants,substr(temp_change,1,1))) &
                             (str_count(temp_OT[[1]],substr(temp_change,3,3
)) > str_count(temp_OT_variants,substr(temp_change,3,3))) &
                             (str_count(temp_OT[[1]],substr(temp_unaffected
,1,1)) == str_count(temp_OT_variants,substr(temp_unaffected,1,1))) &
                             (str_count(temp_OT[[1]],substr(temp_unaffected
,3,3)) == str_count(temp_OT_variants,substr(temp_unaffected,3,3)))),]
    temp_off_target_editing <- colSums(temp_OT[3])
    OT_quantification_samples[k,2] <- temp_off_target_editing
  }
  colnames(OT_quantification_samples) <- c("sample",temp_OT_name)
  OT_quantification <- cbind(OT_quantification, OT_quantification_samples[2])
}
```

```r
# On-Target

for (k in 1:6)
{
  temp_sample_name <-  paste("ON","_",k,".txt",sep = "")
  temp_OT <- read.table(temp_sample_name, skip = 1)
  temp_OT <- temp_OT[,c(1,2,8)]
  temp_OT[,1] <- substr(temp_OT[,1],11,30)
  temp_OT[,2] <- substr(temp_OT[,2],11,30)
  temp_OT_variants <- temp_OT[2,2]
  temp_OT <- temp_OT[which((str_count(temp_OT[[1]],"A") < str_count(temp_OT_v
ariants,"A")) &
                             (str_count(temp_OT[[1]],"G") > str_count(temp_OT
_variants,"G")) &
                             (str_count(temp_OT[[1]],"C") == str_count(temp_O
T_variants,"C")) &
                             (str_count(temp_OT[[1]],"T") == str_count(temp_O
T_variants,"T"))),]
  temp_off_target_editing <- colSums(temp_OT[3])
  OT_quantification_samples[k,2] <- temp_off_target_editing
  colnames(OT_quantification_samples) <- c("sample","ON")
}

OT_quantification_per <- cbind(OT_quantification, OT_quantification_samples[2
])
write.csv(OT_quantification_per, file = "OT_quantification_FA55.csv")
```

a

b

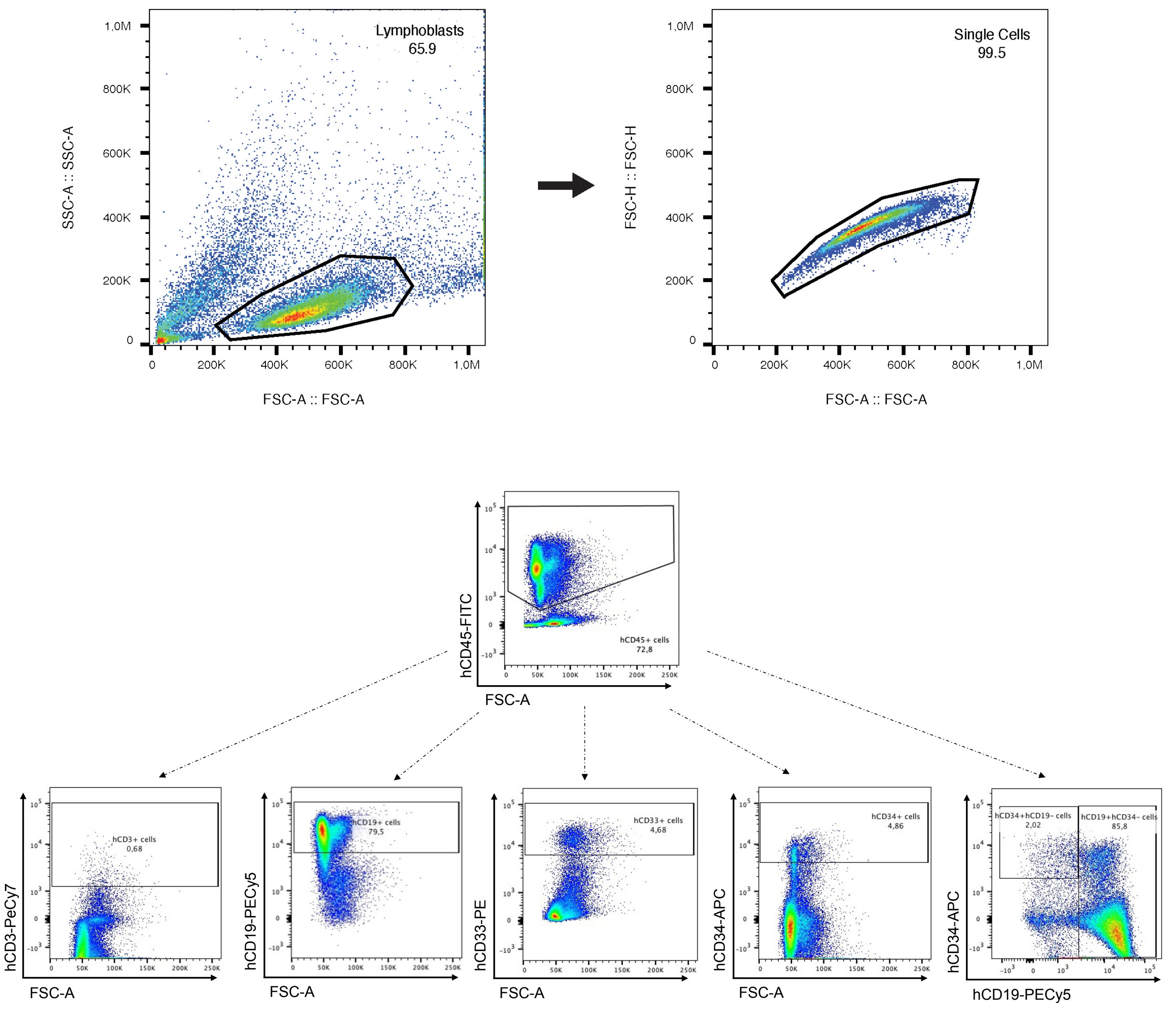

Supplementary Fig. 11: Gating scheme for flow cytometry

(a) The population of live cells were gated on SSC-A vs. FSC-A. To obtain live and single cells we gated on FSC-H vs. FSC-A. This gating strategy was used in Figure 2 and 3 to determine the number of live cells during MMC treatment. (b) Gating used in Figure 5 and Supplementary Figure 6 to determine the different hematopoietic lineages.

Off target id	Genomic location	Intergenic/Intragenic	Gene	Exon/Intron
FA55_OT1	chr6:41,740,360-41,740,379	Intragenic	*PGC*	Intron
FA55_OT2	chr6:71,217,727-71,217,746	Intergenic	NA	NA
FA55_OT3	chr5:153,559,954-153,559,973	Intragenic	*GRIA1*	Intron
FA55_OT4	chr6:39,094,875-39,094,894	Intergenic	NA	NA
FA55_OT5	chr22:20,718,351-20,718,370	Intragenic	*PI4KA*	Intron
FA55_OT6	chr4:17,740,777-17,740,796	Intragenic	*FAM184B*	Intron
FA55_OT7	chr19:1,251,831-1,251,850	Intragenic	*MIDN*	Intron-Exon Junction
FA55_OT8	chr11:54,581,250-54,581,269	Intergenic	NA	NA

Table1A: List of Cas-OFFinder off-target sites for FA-55 guide 1 analyzed in the study

Off target id	Genomic location	Intergenic/Intragenic	Gene	Exon/Intron
FA55_OT9	chr15:94,782,218-94,782,237	Intergenic	NA	NA
FA55_OT10	chr8:6,624,175-6,624,195	Intragenic	MCPH1	Intron
FA55_OT11	chr16:79,610,588-79,610,607	Intergenic	NA	NA
FA55_OT12	chr2:36,704,637-36,704,656	Intragenic	VIT	Intron
FA55_OT13	chr6:110,387,381-110,387,400	Intergenic	NA	NA
FA55_OT14	chr6:111,953,896-111,953,915	Intergenic	NA	NA
FA55_OT15	chrX:73,779,056-73,779,075	Intergenic	NA	NA
FA55_OT16	chr10:85,922,391-85,922,410	Intragenic	GRID1	Intron
FA55_OT17	chr5:21,723,923-21,723,943	Intragenic	ENSG00000253766	Intron
FA55_OT18	chr10:123,703,913-123,703,932	Intergenic	NA	NA
FA55_OT19	chr11:78,671,487-78,671,506	Intragenic	TENM4	Intron
FA55_OT20	chr8:110,176,943-110,176,962	Intergenic	NA	NA
FA55_OT21	chr10:126,631,292-126,631,312	Intragenic	C10orf90	Intron
FA55_OT22	chr1:26,133,068-26,133,087	Intergenic	NA	NA
FA55_OT23	chr2:85,950,441-85,950,461	Intergenic	NA	NA
FA55_OT24	chr9:33,588,065-33,588,084	Intragenic	CYP4F26P	Intron
FA55_OT25	chrX:46,760,017-46,760,036	Intergenic	NA	NA
FA55_OT26	chr5:135,833,782-135,833,802	Intragenic	SLC25A48	Intron
FA55_OT27	chr3:61,488,058-61,488,078	Intergenic	NA	NA
FA55_OT28	chr15:68,017,636-68,017,656	Intergenic	NA	NA
FA55_OT29	chr5:5,623,859-5,623,878	Intergenic	NA	NA
FA55_OT30	chr1:63,916,951-63,916,970	Intragenic	ROR1	Intron
FA55_OT31	chr12:60,842,928-60,842,947	Intergenic	NA	NA
FA55_OT32	chr10:113,549,470-113,549,489	Intergenic	NA	NA
FA55_OT33	chr18:59,570,920-59,570,939	Intragenic	CCBE1	Intron
FA55_OT34	chr3:166,110,116-166,110,134	Intergenic	NA	NA
FA55_OT35	chr1:212,195,811-212,195,830	Intragenic	LINC02608	Intron
FA55_OT36	chr9:31,646,596-31,646,614	Intergenic	NA	NA
FA55_OT37	chr5:33,745,214-33,745,233	Intragenic	ADAMTS12	Intron
FA55_OT38	chr8:48,270,921-48,270,940	Intergenic	NA	NA

Table1B: List of CRISTA off-target sites for FA-55 guide 1 analyzed in the study

Off target id	Genomic location	Intergenic/Intragenic	Gene	Exon/Intron
FA75_OT1	chr2:110,027,307-110,027,506	Intergenic	NA	NA
FA75_OT2	chr2:110,344,019-110,344,038	Intergenic	NA	NA
FA75_OT3	chr2:202,148,657-202,148,676	Intragenic	*KIAA2012*	Intron
FA75_OT4	chr10:14,263,604-14,263,623	Intragenic	*FRMD4A*	Intron
FA75_OT5	chr9:42,184,196-42,184,215	Intragenic	*SPATA31A6*	Intron
FA75_OT6	chr14: 90,091,562-90,091,581	Intragenic	*KCNK13*	Intron
FA75_OT7	chr18:48,679,450-48,679,469	Intragenic	*CTIF*	Intron
FA75_OT8	chr3:63,777,216-63,777,235	Intergenic	NA	NA

Table2A: List of Cas-OFFinder off-target sites for FA-75 guide 4 analyzed in the study

Off target id	Genomic location	Intergenic/Intragenic	Gene	Exon/Intron
FA75_OT9	chr17:64,099,725-64,099,744	Intragenic	*ERN1*	Intron
FA75_OT10	chr17:50,367,093-50,367,112	Intergenic	NA	NA
FA75_OT11	chr16:87,299,215-87,299,234	Intergenic	NA	NA
FA75_OT12	chr6:117,266,257-117,266,276	Intragenic	*VGLL2*	Intron
FA75_OT13	chr6:158,102,075-158,102,094	Intergenic	NA	NA
FA75_OT14	chr11:61,288,777-61,288,796	Intragenic	*VWCE*	Intron
FA75_OT15	chr10:37,265,401-37,265,420	Intergenic	NA	NA
FA75_OT16	chr5:14,206,548-14,206,567	Intragenic	*TRIO*	Intron
FA75_OT17	chr1:36,627,035-36,627,054	Intergenic	NA	NA
FA75_OT18	chr16:72,103,445-72,103,464	Intragenic	*DHX38*	Intron
FA75_OT19	chr1:36,855,294-36,855,313	Intragenic	*GRIK3*	Intron
FA75_OT20	chr10:112,641,890-112,641,911	Intragenic	*VTI1A*	Intron
FA75_OT21	chr2:189,515,498-189,515,517	Intergenic	NA	NA
FA75_OT22	chr15:97,360,120-97,360,139	Intragenic	*LINC02253*	Intron
FA75_OT23	chr3:165,744,530-165,744,549	Intragenic	*LINC01322*	Intron
FA75_OT24	chrX:20,037,534-20,037,553	Intragenic	*MAP7D2*	Intron
FA75_OT25	chr17:34,406,616-34,406,636	Intergenic	NA	NA
FA75_OT26	chr13:61,709,395-61,709,414	Intergenic	NA	NA
FA75_OT27	chr16:31,876,974-31,876,993	Intragenic	*ZNF267*	Intron
FA75_OT28	chr1:151,945,772-151,945,791	Intragenic	*ENSG00000286581*	Intron
FA75_OT29	chr20:60,782,832-60,782,851	Intergenic	NA	NA

Table2B: List of CRISTA off-target sites for FA-75 guide 4 analyzed in the study

Oligonucleotide	Sequence (5' - 3')
sgRNA (1) FA-55 NGG (with cloning overhangs)	TTGGCTTTGTAGGATCAAGCCTCAGTTTAAGAGC
sgRNA (1) FA-55 NGG (with cloning overhangs)	TTAGCTCTTAAACTGAGGCTTGATCCTACAAAGCCAACAAG
sgRNA (4) LFA-75 NGG (with cloning overhangs)	TTGGGCCCAACAGCCTCTTTCTGGTTTAAGAGC
sgRNA (4) LFA-75 NGG (with cloning overhangs)	TTAGCTCTTAAACCAGAAAGAGGCTGTTGGGCCCAACAAG
Synthetic FA-55 sgRNA1 (RNA)	CUUUGUAGGAUCAAGCCUCA
Synthetic LFA-75 sgRNA4 (RNA)	GGCCCAACAGCCUCUUUCUG
NT sgRNA protospacer sequence	GCGCCAAACGTGCCCTGACGG

Primer	Sequence (5' - 3')
FA-55 Sanger sequencing fwd	AGGGGCAGGCTTATCTCTGA
FA-55 Sanger sequencing rev	CGGGCAGGTTTCCTCATCTT
LFA-75 Sanger sequencing fwd	AATCAAACCATCTAAGTGCTGCTG
LFA-75 Sanger sequencing rev	CCGGGTCACTCTCTGTTTCAG
FA-55 NGS fwd	CTTTCCCTACACGACGCTCTTCCGATCTGCTATGGTTTTGTTTTGTGTTTAAGGC
FA-55 NGS rev	GGAGTTCAGACGTGTGCTCTTCCGATCTAACCATCCCGGCTGAGAGA
LFA-75 NGS fwd	CTTTCCCTACACGACGCTCTTCCGATCTAAACGGCCGGGTCACTCTCT
LFA-75 NGS rev	GGAGTTCAGACGTGTGCTCTTCCGATCTAAGGTGCAAGGGTCTCCAG
ABEmax/ABE8e HIFI assembly (1)	ATGTCCGGACTCAGATCTCGAAAACGGACAGCCGACGGAAG
ABEmax/ABE8e HIFI assembly (2)	TCGAGATCTGAGTCCGGACATGGTGGGCGGCCGCATTG
ABEmax/ABE8e HIFI assembly (3)	GGAAAGCGAGTTCGTGTACGGC
ABEmax/ABE8e HIFI assembly (4)	TACACGAACTCGCTTTCCAGCTTAGG
ABEmax/ABE8e HIFI assembly (5)	GGATCCACCGGATCTAGATAACTGATCG
ABEmax/ABE8e HIFI assembly (6)	TCTAGATCCGGTGGATCCGACTTTCCTCTTCTTCTTGGGCTCGAATT
LFA-75 OT1 NGS fwd	CTTTCCCTACACGACGCTCTTCCGATCTACATTGGTTCTCCCACCAGC
LFA-75 OT1 NGS rev	GGAGTTCAGACGTGTGCTCTTCCGATCTCACAGCCACAGGGGAAAAGT
LFA-75 OT2 NGS fwd	CTTTCCCTACACGACGCTCTTCCGATCTAAAAGTGCCCTGCTGGCT
LFA-75 OT2 NGS rev	GGAGTTCAGACGTGTGCTCTTCCGATCTGCGGTTACATGTTCACCCTCA
LFA-75 OT3 NGS fwd	CTTTCCCTACACGACGCTCTTCCGATCTCATTTGTGTGGGTGGAAGCC
LFA-75 OT3 NGS rev	GGAGTTCAGACGTGTGCTCTTCCGATCTTCTGTCTAGGTTTCCCTGGCT
LFA-75 OT4 NGS fwd	CTTTCCCTACACGACGCTCTTCCGATCTTACCCTTGCCCACGATCAG
LFA-75 OT4 NGS rev	GGAGTTCAGACGTGTGCTCTTCCGATCTAAGGAGCTTAGAGGGCCCA
LFA-75 OT5 NGS fwd	CTTTCCCTACACGACGCTCTTCCGATCTAGGTCTCTGTCCGAGACCAG
LFA-75 OT5 NGS rev	GGAGTTCAGACGTGTGCTCTTCCGATCTGAAGACCAGAGGAGACACCAG
LFA-75 OT6 NGS fwd	CTTTCCCTACACGACGCTCTTCCGATCTGCCAGTTGACACAGCACAG
LFA-75 OT6 NGS rev	GGAGTTCAGACGTGTGCTCTTCCGATCTTAATGAGGCATTTGGCATGCC
LFA-75 OT7 NGS fwd	CTTTCCCTACACGACGCTCTTCCGATCTACATCTGGAAGCAGGCAGAG
LFA-75 OT7 NGS rev	GGAGTTCAGACGTGTGCTCTTCCGATCTAGGTCTCAGTCTCAGGAGGAG
LFA-75 OT8 NGS fwd	CTTTCCCTACACGACGCTCTTCCGATCTCATTACCCAGTCTCAGGT
LFA-75 OT8 NGS rev	GGAGTTCAGACGTGTGCTCTTCCGATCTCATATTTATTGGCCCAACTG
LFA-75 OT9 NGS fwd	CTTTCCCTACACGACGCTCTTCCGATCTATTTGTGCATGGGAGCCTGATC
LFA-75 OT9 NGS rev	GGAGTTCAGACGTGTGCTCTTCCGATCTTGTTTCACTCCCTCCGTTGGT
LFA-75 OT10 NGS fwd	CTTTCCCTACACGACGCTCTTCCGATCTTCAAGGCAGAACTCCAGAACT
LFA-75 OT10 NGS rev	GGAGTTCAGACGTGTGCTCTTCCGATCTGGGCCGGGAAGAAAATCTCAT
LFA-75 OT11 NGS fwd	CTTTCCCTACACGACGCTCTTCCGATCTCACACCTCCCACCCATCATC
LFA-75 OT11 NGS rev	GGAGTTCAGACGTGTGCTCTTCCGATCTAGGCACACATGAACACAAGGA
LFA-75 OT12 NGS fwd	CTTTCCCTACACGACGCTCTTCCGATCTGCAAAGCTGACGCAGGAAAAA
LFA-75 OT12 NGS rev	GGAGTTCAGACGTGTGCTCTTCCGATCTCGCCAGAGAATTGAAACGGAAG
LFA-75 OT13 NGS fwd	CTTTCCCTACACGACGCTCTTCCGATCTCTGTGTGGTAGAATGGTTAAGGT
LFA-75 OT13 NGS rev	GGAGTTCAGACGTGTGCTCTTCCGATCTCTAATTCGGGAAGCAAGAGCA
LFA-75 OT14 NGS fwd	CTTTCCCTACACGACGCTCTTCCGATCTTGTCCCAAGGTGAAAGGCATT
LFA-75 OT14 NGS rev	GGAGTTCAGACGTGTGCTCTTCCGATCTAAAAACGCGCCATAAAGAGGC
LFA-75 OT15 NGS fwd	CTTTCCCTACACGACGCTCTTCCGATCTGGTCCATGCCTCCTCAATTTAC
LFA-75 OT15 NGS rev	GGAGTTCAGACGTGTGCTCTTCCGATCTAAGACTGGCTGTGTGCATCA
LFA-75 OT16 NGS fwd	CTTTCCCTACACGACGCTCTTCCGATCTGGACTTTGCATCCTCTGAACTCA
LFA-75 OT16 NGS rev	GGAGTTCAGACGTGTGCTCTTCCGATCTTAACACACAGGCACCATCGAA
LFA-75 OT17 NGS fwd	CTTTCCCTACACGACGCTCTTCCGATCTCTGGTAGAGGAAGAGGGAGAGG
LFA-75 OT17 NGS rev	GGAGTTCAGACGTGTGCTCTTCCGATCTCATGGAGAGGGGAGCAAGATG
LFA-75 OT18 NGS fwd	CTTTCCCTACACGACGCTCTTCCGATCTCACTGCACAAAGTAACGGGATG
LFA-75 OT18 NGS rev	GGAGTTCAGACGTGTGCTCTTCCGATCTTCCAAGAAGACTCCGCTACCT
LFA-75 OT19 NGS fwd	CTTTCCCTACACGACGCTCTTCCGATCTGGGCAATGGAAGGTCTGAGTA
LFA-75 OT19 NGS rev	GGAGTTCAGACGTGTGCTCTTCCGATCTAACTGTGGACCCCTGACCTT
LFA-75 OT20 NGS fwd	CTTTCCCTACACGACGCTCTTCCGATCTCCACATCAAGTTTCCAGTCCA
LFA-75 OT20 NGS rev	GGAGTTCAGACGTGTGCTCTTCCGATCTAAAGAGTGGTCTGGGTTGAGA
LFA-75 OT21 NGS fwd	CTTTCCCTACACGACGCTCTTCCGATCTCCAACTGTGGTATGCTCTGCTA
LFA-75 OT21 NGS rev	GGAGTTCAGACGTGTGCTCTTCCGATCTAGTGCTCCCTGTATTTTGGTCA
LFA-75 OT22 NGS fwd	CTTTCCCTACACGACGCTCTTCCGATCTAAACCCCTCACCAAAAACACC
LFA-75 OT22 NGS rev	GGAGTTCAGACGTGTGCTCTTCCGATCTGAATACTCCAGCCAATGCTCAC
LFA-75 OT23 NGS fwd	CTTTCCCTACACGACGCTCTTCCGATCTACTACCTTCTCTCCCAGCCT
LFA-75 OT23 NGS rev	GGAGTTCAGACGTGTGCTCTTCCGATCTACTGCCCAAAGACCTCCTATC
LFA-75 OT24 NGS fwd	CTTTCCCTACACGACGCTCTTCCGATCTACAGATGGCTGTGGATGATATGC
LFA-75 OT24 NGS rev	GGAGTTCAGACGTGTGCTCTTCCGATCTTCTCCTTCCAAGTAAAGCGCA
LFA-75 OT25 NGS fwd	CTTTCCCTACACGACGCTCTTCCGATCTTCCTCCTCTTTTCCACTCCAC
LFA-75 OT25 NGS rev	GGAGTTCAGACGTGTGCTCTTCCGATCTGGTAGGTCACAATGGCACTCT
LFA-75 OT26 NGS fwd	CTTTCCCTACACGACGCTCTTCCGATCTTCTAACCCTTACCTGTGGTCTCA
LFA-75 OT26 NGS rev	GGAGTTCAGACGTGTGCTCTTCCGATCTTTCTGCTGACTGGGACAATTACA
LFA-75 OT27 NGS fwd	CTTTCCCTACACGACGCTCTTCCGATCTAGCCCAGTTAGTTCTTCCAGG
LFA-75 OT27 NGS rev	GGAGTTCAGACGTGTGCTCTTCCGATCTCAACCCTCCTTCCATCCCAAC
LFA-75 OT28 NGS fwd	CTTTCCCTACACGACGCTCTTCCGATCTTGGCTCTCCCCTTTTTCTCTTT
LFA-75 OT28 NGS rev	GGAGTTCAGACGTGTGCTCTTCCGATCTCAGCATCTGCCCTCACGTAG
LFA-75 OT29 NGS fwd	CTTTCCCTACACGACGCTCTTCCGATCTATGACCTGACTCTTACCCTGGA
LFA-75 OT29 NGS rev	GGAGTTCAGACGTGTGCTCTTCCGATCTTGTTGTTTCCGTGTGTGTGTTC
FA-55 OT1 NGS fwd	CTTTCCCTACACGACGCTCTTCCGATCTTCCACTCTCTTCTCCAGGCT
FA-55 OT1 NGS rev	GGAGTTCAGACGTGTGCTCTTCCGATCTTGGCACTGCTCTGGGATG
FA-55 OT2 NGS fwd	CTTTCCCTACACGACGCTCTTCCGATCTCAGTGACTGGAACCTGGCTT
FA-55 OT2 NGS rev	GGAGTTCAGACGTGTGCTCTTCCGATCTTCTGTGTTGATGGGGTGGTC
FA-55 OT3 NGS fwd	CTTTCCCTACACGACGCTCTTCCGATCTACACACCCTATTGCCTCCTTC
FA-55 OT3 NGS rev	GGAGTTCAGACGTGTGCTCTTCCGATCTTTCAGAGTAAACTTTGTAAACAGCA
FA-55 OT4 NGS fwd	CTTTCCCTACACGACGCTCTTCCGATCTGCAGCAAGATCACAGAAACCT
FA-55 OT4 NGS rev	GGAGTTCAGACGTGTGCTCTTCCGATCTCATGATCATTTTGGAAATTCATGTTAGAATCT
FA-55 OT5 NGS fwd	CTTTCCCTACACGACGCTCTTCCGATCTACGATGACAGGACCTGCAAA
FA-55 OT5 NGS rev	GGAGTTCAGACGTGTGCTCTTCCGATCTCAATTTGAGGCCACCCTGC
FA-55 OT6 NGS fwd	CTTTCCCTACACGACGCTCTTCCGATCTAGAAGCCAACCTCCAGGCCC
FA-55 OT6 NGS rev	GGAGTTCAGACGTGTGCTCTTCCGATCTCGACAGTGAAACCTGTTGTGATGGAA
FA-55 OT7 NGS fwd	CTTTCCCTACACGACGCTCTTCCGATCTGATTCCGACCCCACACTCAG
FA-55 OT7 NGS rev	GGAGTTCAGACGTGTGCTCTTCCGATCTGAGAGCTTGCATCACGGACT
FA-55 OT8 NGS fwd	CTTTCCCTACACGACGCTCTTCCGATCTATGACACCATCCATAACCTATACACATATATT
FA-55 OT8 NGS rev	GGAGTTCAGACGTGTGCTCTTCCGATCTCATTTGACCCAGAATTTTCACCCTT
FA-55 OT9 NGS fwd	CTTTCCCTACACGACGCTCTTCCGATCTGAAGAGAAAGCAAATGGGGAAGA
FA-55 OT9 NGS rev	GGAGTTCAGACGTGTGCTCTTCCGATCTTAAGTTGGGTTGAGAATGGATTGG
FA-55 OT10 NGS fwd	CTTTCCCTACACGACGCTCTTCCGATCTACTTCCTCCGGTTGTTTTGC
FA-55 OT10 NGS rev	GGAGTTCAGACGTGTGCTCTTCCGATCTGGACACAAGTTACAGGAGGAAAG
FA-55 OT11 NGS fwd	CTTTCCCTACACGACGCTCTTCCGATCTCATCTCTCCATCTCCTTCACCT
FA-55 OT11 NGS rev	GGAGTTCAGACGTGTGCTCTTCCGATCTGGTAAGAAGTGCTGTGTTGCA
FA-55 OT12 NGS fwd	CTTTCCCTACACGACGCTCTTCCGATCTAAGTAGGACATTAGGTGGTTGCC
FA-55 OT12 NGS rev	GGAGTTCAGACGTGTGCTCTTCCGATCTAATCCCAGCAACCAGAGACCT
FA-55 OT13 NGS fwd	CTTTCCCTACACGACGCTCTTCCGATCTGGTTTCAGTTTCAGAGCCCAA
FA-55 OT13 NGS rev	GGAGTTCAGACGTGTGCTCTTCCGATCTAAGGACAGTTCCCAGAAGGTT
FA-55 OT14 NGS fwd	CTTTCCCTACACGACGCTCTTCCGATCTCCTCTCTACTCTGCCCTGTTTT
FA-55 OT14 NGS rev	GGAGTTCAGACGTGTGCTCTTCCGATCTCGGTGTCCAGGCAATAAATGTT
FA-55 OT15 NGS fwd	CTTTCCCTACACGACGCTCTTCCGATCTTACCTGTTATTCTGCCGTCTTGT
FA-55 OT15 NGS rev	GGAGTTCAGACGTGTGCTCTTCCGATCTGAGTGTTCTGATGATGTTCCATAGT
FA-55 OT16 NGS fwd	CTTTCCCTACACGACGCTCTTCCGATCTCTCATAAGCCTGAAACCATTATTCC
FA-55 OT16 NGS rev	GGAGTTCAGACGTGTGCTCTTCCGATCTGGATGCCTAGATAAGTGGTCAAC
FA-55 OT17 NGS fwd	CTTTCCCTACACGACGCTCTTCCGATCTTGTGTAAGAACAGGGAATAGGGA
FA-55 OT17 NGS rev	GGAGTTCAGACGTGTGCTCTTCCGATCTGAAGAGAGAGGAAAGGCATAAAGT
FA-55 OT18 NGS fwd	CTTTCCCTACACGACGCTCTTCCGATCTATCCCTGACCCCCACCCTTAT
FA-55 OT18 NGS rev	GGAGTTCAGACGTGTGCTCTTCCGATCTTGCACTCACCCCACTTACGT
FA-55 OT19 NGS fwd	CTTTCCCTACACGACGCTCTTCCGATCTTCAGAGCAAGCACAGCCTAT
FA-55 OT19 NGS rev	GGAGTTCAGACGTGTGCTCTTCCGATCTCCAGGGGTAAGAATCAGAAATGA
FA-55 OT20 NGS fwd	CTTTCCCTACACGACGCTCTTCCGATCTCCTGAAGAAACTCCAAGCAAAC
FA-55 OT20 NGS rev	GGAGTTCAGACGTGTGCTCTTCCGATCTTCAAGAAAGCTAGAAAGTGCAG
FA-55 OT21 NGS fwd	CTTTCCCTACACGACGCTCTTCCGATCTGCAAAACGAAGCCTGTCACT
FA-55 OT21 NGS rev	GGAGTTCAGACGTGTGCTCTTCCGATCTTTGAGGAAGGAACGGGACTG
FA-55 OT22 NGS fwd	CTTTCCCTACACGACGCTCTTCCGATCTAACCCTCCTGGGCTAAACTCC
FA-55 OT22 NGS rev	GGAGTTCAGACGTGTGCTCTTCCGATCTACTGGCCCTCCATCACCTTTT
FA-55 OT23 NGS fwd	CTTTCCCTACACGACGCTCTTCCGATCTTCCCAGTGACGAAAGCCAATAAG
FA-55 OT23 NGS rev	GGAGTTCAGACGTGTGCTCTTCCGATCTAATGTGGTGTGGAAAGAGGTAGC
FA-55 OT24 NGS fwd	CTTTCCCTACACGACGCTCTTCCGATCTGGGTAGTAGGAAGGGGGAGTA
FA-55 OT24 NGS rev	GGAGTTCAGACGTGTGCTCTTCCGATCTCAGATTCATAGACCCACCAACAC
FA-55 OT25 NGS fwd	CTTTCCCTACACGACGCTCTTCCGATCTACCCTGATGACCCTTTCTTTTATG
FA-55 OT25 NGS rev	GGAGTTCAGACGTGTGCTCTTCCGATCTTCTGATCTTGTTTCTTCCTTGACC
FA-55 OT26 NGS fwd	CTTTCCCTACACGACGCTCTTCCGATCTCTCTCCCGATACAGGTCCAC
FA-55 OT26 NGS rev	GGAGTTCAGACGTGTGCTCTTCCGATCTCATGTCACGTCATCCAGGTTAAT
FA-55 OT27 NGS fwd	CTTTCCCTACACGACGCTCTTCCGATCTTGAGTTCACACTCCATTTAGCC
FA-55 OT27 NGS rev	GGAGTTCAGACGTGTGCTCTTCCGATCTGAGAGATACAGGAGATGTGAAAATG
FA-55 OT28 NGS fwd	CTTTCCCTACACGACGCTCTTCCGATCTGCGCCACCTTGATGAGATAT
FA-55 OT28 NGS rev	GGAGTTCAGACGTGTGCTCTTCCGATCTTCACAAAACCCACACCACTATC
FA-55 OT29 NGS fwd	CTTTCCCTACACGACGCTCTTCCGATCTTCACCATCATATCGCACCCTCT
FA-55 OT29 NGS rev	GGAGTTCAGACGTGTGCTCTTCCGATCTGCAAGCTCCTTTGATTCCATGTC
FA-55 OT30 NGS fwd	CTTTCCCTACACGACGCTCTTCCGATCTTTTCTCCTATAAAGCCCTGGCC
FA-55 OT30 NGS rev	GGAGTTCAGACGTGTGCTCTTCCGATCTAGAATTGAAAGCTGAAGGGGAAG
FA-55 OT31 NGS fwd	CTTTCCCTACACGACGCTCTTCCGATCTCCAGCAGCCACAGAAGTCATATA
FA-55 OT31 NGS rev	GGAGTTCAGACGTGTGCTCTTCCGATCTCACAGTAACGAATTATCTCCCAAATCC
FA-55 OT32 NGS fwd	CTTTCCCTACACGACGCTCTTCCGATCTCGGAGGCCCAGAAAGGATAAAT
FA-55 OT32 NGS rev	GGAGTTCAGACGTGTGCTCTTCCGATCTGTGTTTCACGTTTGTTTTCCTCAAG
FA-55 OT33 NGS fwd	CTTTCCCTACACGACGCTCTTCCGATCTAGCCAAGGAATCAGAGCAGAAT
FA-55 OT33 NGS rev	GGAGTTCAGACGTGTGCTCTTCCGATCTCATACGTTACAACTCAGCAGTCG
FA-55 OT34 NGS fwd	CTTTCCCTACACGACGCTCTTCCGATCTACACCTACTCAGGCTTTTCTCT
FA-55 OT34 NGS rev	GGAGTTCAGACGTGTGCTCTTCCGATCTTATGGGAGGTAGAGGAAAAGGG
FA-55 OT35 NGS fwd	CTTTCCCTACACGACGCTCTTCCGATCTTAGTGGAGCTGTGAGAAGGCA
FA-55 OT35 NGS rev	GGAGTTCAGACGTGTGCTCTTCCGATCTCTTTGAATCCATGTCCCACACC
FA-55 OT36 NGS fwd	CTTTCCCTACACGACGCTCTTCCGATCTCAGGTTTAGAGGGAGGTAGAAGA
FA-55 OT36 NGS rev	GGAGTTCAGACGTGTGCTCTTCCGATCTTTTGCATCTGCCTCTCCTTTG
FA-55 OT37 NGS fwd	CTTTCCCTACACGACGCTCTTCCGATCTGATCTTTGGGGGTTTTCTTTAGAGT
FA-55 OT37 NGS rev	GGAGTTCAGACGTGTGCTCTTCCGATCTAGTTTTCCCTTCATCATTCCTGTG
FA-55 OT38 NGS fwd	CTTTCCCTACACGACGCTCTTCCGATCTGAAAACAGGTCAGGAGAGGTC
FA-55 OT38 NGS rev	GGAGTTCAGACGTGTGCTCTTCCGATCTTGCCAGATAAATCCGTAACATG

Table3: List of oligos and primers used in the study
