# Supplementary material for: Adenine base editing efficiently restores the function of Fanconi anemia hematopoietic stem and progenitor cells
Source: Nat Commun. 2022 Nov 12;13:6900. doi: 10.1038/s41467-022-34479-z (PMC9653444; doi:10.1038/s41467-022-34479-z)
Supplement: Supplementary file 3 — Reporting Summary [file 41467_2022_34479_MOESM3_ESM.pdf]

nature portfolio

Corresponding author(s): Mehmet E. Karasu, Paula Rio,Jacob E. Corn

Last updated by author(s): Otober 18, 2022

Reporting Summary

Nature Portfolio wishes to improve the reproducibility of the work that we publish. This form provides structure for consistency and transparency in reporting. For further information on Nature Portfolio policies, see our Editorial Policies and the Editorial Policy Checklist.

Statistics

For all statistical analyses, confirm that the following items are present in the figure legend, table legend, main text, or Methods section.

n/a	Confirmed	
☐	☒	The exact sample size (*n*) for each experimental group/condition, given as a discrete number and unit of measurement
☐	☒	A statement on whether measurements were taken from distinct samples or whether the same sample was measured repeatedly
☐	☒	The statistical test(s) used AND whether they are one- or two-sided<br>*Only common tests should be described solely by name; describe more complex techniques in the Methods section.*
☒	☐	A description of all covariates tested
☒	☐	A description of any assumptions or corrections, such as tests of normality and adjustment for multiple comparisons
☐	☒	A full description of the statistical parameters including central tendency (e.g. means) or other basic estimates (e.g. regression coefficient) AND variation (e.g. standard deviation) or associated estimates of uncertainty (e.g. confidence intervals)
☐	☒	For null hypothesis testing, the test statistic (e.g. *F*, *t*, *r*) with confidence intervals, effect sizes, degrees of freedom and *P* value noted<br>*Give P values as exact values whenever suitable.*
☒	☐	For Bayesian analysis, information on the choice of priors and Markov chain Monte Carlo settings
☒	☐	For hierarchical and complex designs, identification of the appropriate level for tests and full reporting of outcomes
☒	☐	Estimates of effect sizes (e.g. Cohen's *d*, Pearson's *r*), indicating how they were calculated

Our web collection on statistics for biologists contains articles on many of the points above.

Software and code

Policy information about availability of computer code

Data collection	Flow cytometry: BD FACSDiva™ Software, Attune NxT Flow Cytometer (with autosampler)
Data analysis	FACS - flowjo (v10.7.1);Illumina amplicon sequencing: Crispresso2 (Clement et al. 2019), Off target analysis: Cas-OFFinder (http://www.rgenome.net/cas-offinder/) 2.4.1, Statistical analysis: GraphPad Prism 8.3.1

For manuscripts utilizing custom algorithms or software that are central to the research but not yet described in published literature, software must be made available to editors and reviewers. We strongly encourage code deposition in a community repository (e.g. GitHub). See the Nature Portfolio guidelines for submitting code & software for further information.

Data

Policy information about availability of data

All manuscripts must include a data availability statement. This statement should provide the following information, where applicable:

- Accession codes, unique identifiers, or web links for publicly available datasets
- A description of any restrictions on data availability
- For clinical datasets or third party data, please ensure that the statement adheres to our policy

Sequencing data is deposited in SRA BioProject PRJNA891670. Link: https://www.ncbi.nlm.nih.gov/sra/PRJNA891670

March 2021

Field-specific reporting

Please select the one below that is the best fit for your research. If you are not sure, read the appropriate sections before making your selection.

☒ Life sciences ☐ Behavioural & social sciences ☐ Ecological, evolutionary & environmental sciences

For a reference copy of the document with all sections, see nature.com/documents/nr-reporting-summary-flat.pdf

Life sciences study design

All studies must disclose on these points even when the disclosure is negative.

Sample size	No sample size calculations were performed. Sample size was determined to be adequate based on the consistency of measurable differences and previously published literature in the genome editing field.
Data exclusions	Data were excluded from analysis in the case of failed experiments due to factors such as loss of reagent activity, contamination.
Replication	Each data point represents an individual biological replicate as listed in figure legends and all replications were successful.
Randomization	No randomization was performed. All independent biological experiments were treated equally.
Blinding	No blinding was performed since the data is not subjective.

Reporting for specific materials, systems and methods

We require information from authors about some types of materials, experimental systems and methods used in many studies. Here, indicate whether each material, system or method listed is relevant to your study. If you are not sure if a list item applies to your research, read the appropriate section before selecting a response.

Materials & experimental systems

n/a	Involved in the study	
☐	☒	Antibodies
☐	☒	Eukaryotic cell lines
☒	☐	Palaeontology and archaeology
☐	☒	Animals and other organisms
☐	☒	Human research participants
☒	☐	Clinical data
☒	☐	Dual use research of concern

Methods

n/a	Involved in the study	
☒	☐	ChIP-seq
☐	☒	Flow cytometry
☒	☐	MRI-based neuroimaging

Antibodies

Antibodies used	anti-rabbit FANCA antibody (ab5036 (Abcam) or A301-980A (bethyl), anti-rabbit FANCD2 antibody (ab221932, (Abcam)), anti-goat HSP60 antibody (sc-1052, Santa Cruz Biotechnology), with anti-rabbit secondary antibody (IRDye 800CW (926-32213) or anti-goat IRDye 800CW (926-32214)<br>anti-hCD3-PE, Beckman Coultek Nº Cat A07775<br>anti-hCD19-PE, Beckman Coultek Nº Cat IM1285U<br>anti-hCD-235a PE, BD Pharmingen Nº Cat 554489<br>anti-hCD45-FITC, BioLegend Nº Cat 304006<br>anti-hCD3-PECy7, BioLegend Nº Cat 300419<br>anti-hCD19-PECy5, BioLegend Nº Cat 302209<br>antih-hCD33-PE, eBioscience Nº Cat 12-0339-42<br>antih-hCD34-APC, BD Biosciences Nº Cat 345804<br>anti-hCD14-PE, BD Pharmingen Cat Nº 345785<br>anti-hCD15-PE, Beckman Coultek, Cat Nº IM1954U
Validation	No further validation is performed for the commercial antibodies which are highly used in different publications . Except, appropriate controls (FA-55 LCL extract) were used to confirm the antibody performance for anti-rabbit FANCA antibody.

Eukaryotic cell lines

Policy information about cell lines

Cell line source(s)	Healthy Donor, FA-55, FA-75 lymphoblastic cell lines are obtained from CIEMAT and Jordi Surralles' lab (Universidad Autónoma de Barcelona,) Spain
Authentication	Sequencing
Mycoplasma contamination	All cell lines were routinely tested for mycoplasma (MycoAlert; Lonza) and tested negatively.
Commonly misidentified lines (See ICLAC register)	No commonly misidentified lines were used in this study.

Animals and other organisms

Policy information about studies involving animals; ARRIVE guidelines recommended for reporting animal research

Laboratory animals	Species: non-obese diabetic (NOD) immunodeficient Cg-Prkdcscid Il2rgtm1Wjl/SzJ mice (NSG)<br>Sex: Female<br>Number of mice used for transplantation studies<br>CB primary recipients: 7 mice (mock), 7 mice (ABEmax) and 6 mice (ABE8e). Total number of mice: 20. Sex: Female. Age: 11 weeks (Figure 5D and supplementary figure 6F).<br>CB secondary recipients 7 mice (mock), 7 mice (ABEmax) and 6 mice (ABE8e). Total number of mice: 20 . Sex: Female. Age: 9 weeks (Figure 5E and supplementary figure 6G).<br>mPB primary recipients 7 mice (mock), 6 mice(ABEmax) and 6 mice (ABE8e). Total number of mice: 19. Sex: Female. Age: 11 weeks (Figure 5G and supplementary figure 6H).
Wild animals	NA
Field-collected samples	NA
Ethics oversight	All experimental procedures were conducted according to European and Spanish regulations (European convention ETS 123, regarding the use and protection of vertebrate mammals used in experimentation and other scientific purposes, Directive 2010/63/UE, Spanish Law 6/2013 and Real Decreto (R.D.) 53/2013 regarding the protection and use of animals in scientific research). Procedures involving Genetically Modified Organisms were conducted according to the proper European and Spanish regulations (Directive 2009/41/CE, Spanish Law 9/2003 and R.D. 178/2004). Procedures were approved by the CIEMAT Animal Experimentation Ethical Committee according to all external and internal biosafety and bioethics guidelines, and authorized by the Comunidad de Madrid Government (Codes: PROEX #070-15 and PROEX #156.5/21 Cell and Gene Therapy in rare diseases with chromosomal instability).

Note that full information on the approval of the study protocol must also be provided in the manuscript.

Human research participants

Policy information about studies involving human research participants

Population characteristics	This paper does not include studies involving human research participants, only samples obtained from human participants. In this respect, HD CB derived cells from male and female were used (Figure 5B, D, E and F and supplementary figure 6A, B, D, F and G).<br>HD mPB were obtained from n=4 female donors and n=1 male donor aged 14-39 (Figure 5C, Figure 5G and H and supplementary figure 6C, E and H) and FA patients mPB cells were obtained from the negative fraction discarded after CD34+ selection in the FANCOSTEM trial (Eudra number CT 2011-006197-88) (Figure 6 and supplementary figure 7). FA-A patients age: under 5 years.
Recruitment	HD CB CD34+cells were obtained upon approval by the Centro de Transfusiones de la Comunidad de Madrid (Madrid) and after informed consent was signed. The use of human mPB samples has been approved by the Ethics Comittee at Hospital Infantil Universitario Niño Jesús (Madrid) (Ref: 07/029193.9/21). In this respect, HD CB derived cells from male and female were used. HD mPB were obtained from n=4 female donors and n=1 male donor aged 14-39. mPB samples from FA-A patients under 5 years were obtained from the discarded negative fraction after CD34+ selection in the FANCOSTEM trial (Eudra number CT 2011-006197-88). In all cases participants or legal representatives were informed in detail about the research purpose and informed consent was signed.<br>In no case participants have received any compensation for their participation in these studies.
Ethics oversight	HD CB CD34+cells were obtained upon approval by the Centro de Transfusiones de la Comunidad de Madrid (Madrid) and after informed consent was signed. The use of human mPB samples has been approved by the Ethics Comittee at Hospital Infantil Universitario Niño Jesús (Madrid) (Ref: 07/029193.9/21). In this respect, HD CB derived cells from male and female

were used. HD mPB were obtained from n=4 female donors and n=1 male donor aged 14-39. mPB samples from FA-A patients under 5 years were obtained from the discarded negative fraction after CD34+ selection in the FANCOSTEM trial (Eudra number CT 2011-006197-88). In all cases participants or legal representatives were informed in detail about the research purpose and informed consent was signed.
In no case participants have received any compensation for their participation in these studies.

Note that full information on the approval of the study protocol must also be provided in the manuscript.

Flow Cytometry

Plots

Confirm that:

- [x] The axis labels state the marker and fluorochrome used (e.g. CD4-FITC).
- [x] The axis scales are clearly visible. Include numbers along axes only for bottom left plot of group (a 'group' is an analysis of identical markers).
- [x] All plots are contour plots with outliers or pseudocolor plots.
- [x] A numerical value for number of cells or percentage (with statistics) is provided.

Methodology

Sample preparation

For MMC survival, cells were resuspended in PBS/5% FBS and placed in 96 well plated and analyzed immediately,
For human HSPCs, Human CD34+ HSPCs were purified from the mononuclear fraction by immunoselection using the CD34 Micro-Bead Kit (MACS, Miltenyi Biotec). Magnetic-labelled cells were selected with a LS colum in QuadroMACSTM Separator (Miltenyi Biotec) following manufacturer's instructions, Purified hCD34+ were then analysed by flow cytometry to evaluate their purity in LSRFortessa Cell Analyser (BD). Purities ranging from 85-98% were routinely obtained.
For HSPCs transplantation experiment,30 and 60 days after transplantation, bone marrow samples were obtained by intra-femoral aspiration and total human engraftment was measured by flow cytometry, analysing percentage of hCD45+ cells (anti-hCD45-FITC, BioLengend). Multilineage reconstitution was also evaluated using antibodies against hCD34 (anti-hCD34-APC, BD for HSPCs, hCD33 (anti-hCD33-PE, eBioscience) for myeloid cells, hCD19 (anti-hCD19-Pe-Cy5, BioLegend) for B cells and hCD3 (anti-hCD3-Pe-Cy7, BioLegend) for T cells
Lineage negative populations from FA patients were obtained from apheresis aliquots by the incubation of cells with anti-hCD3-PE, anti-hCD19-PE, anti-hCD33-PE, antih-CD-235a PE for 30 min. Then cells were washed and incubated with anti-PE Microbeads (Miltenyi Biotec). Lineage negative population was confirmed in LSRFortessa Cell Analyser (BD) using FlowJo Software v10.7.1. Cells were grown and cultured during 24 hours prior electroporation in GMP Stem Cell Grow Medium (CellGenix) supplemented with 1% GlutaMAX™ (Gibco), 1% P/S (Gibco), 100 ng/mL SCF and Flt3, 20 ng/mL TPO and IL3 (EuroBiosciences), 10 µg/mL anti-TNFα (Enbrel-Etanercept, Pfizer) and 1 mM N-acetylcysteine (Pharmazam) under hypoxic conditions (37oC, 5% of O2, 5% of CO2 and 95% RH).

Instrument

Attune NxT Flow Cytometer (with autosampler), Thermo Fisher or LSRFortessa Cell Analyser (BD)

Software

FlowJo Software v10.7.1

Cell population abundance

For MMC experiments, we analysed a fixed volume of cell suspension , wild type samples at least containing > 50.000 single events.

Gating strategy

For MMC experiments, cells were first gated for live cells (SSC-A vs FSC-A), then gated for single cells (FSC-H vs FSC-A) to get counts of live cells.
For mouse experiments cells were first gated for live cells (SSC-A vs FSC-A), then gated for single cells (FSC-H vs FSC-A), later on with DAPI to select alive cells, human CD45+ cells were selected and the different subpopulations analysed in the hCD45 population.

- [x] Tick this box to confirm that a figure exemplifying the gating strategy is provided in the Supplementary Information.
